# Nacre-mimetic composite with intrinsic self-healing and shape-programming capability

**DOI:** 10.1038/s41467-019-08643-x

**Published:** 2019-02-18

**Authors:** Gaolai Du, Anran Mao, Jinhong Yu, Jingjing Hou, Nifang Zhao, Jingkai Han, Qian Zhao, Weiwei Gao, Tao Xie, Hao Bai

**Affiliations:** 1 State Key Laboratory of Chemical Engineering, College of Chemical and Biological Engineering, Zhejiang University, 310027 Hangzhou, China; 2 Department of Polymer Science and Engineering, Zhejiang University, 310027 Hangzhou, China

## Abstract

Replicating nacre’s multiscale architecture represents a promising approach to design artificial materials with outstanding rigidity and toughness. It is highly desirable yet challenging to incorporate self-healing and shape-programming capabilities into nacre-mimetic composites due to their rigidity and high filler content. Here, we report such a composite obtained by infiltrating a thermally switchable Diels-Alder network polymer into a lamellar scaffold of alumina. The chemical bond switchability and the physical confinement by the filler endows the composite with sufficient molecular mobility without compromising its thermal dimension stability. Consequently, our composite is capable of self-healing internal damages. Additionally, in contrast to the intractable planar shape of other artificial nacres, precise control of the polymer chain dynamics allows the shape of our composite to be programmed permanently via plasticity and temporarily via shape memory effect. Our approach paves a new way for designing durable multifunctional bioinspired structural materials.

## Abstract (web summary)

Incorporation of self-healing and shape programming capabilities into rigid, structural composite is highly desirable yet challenging. Here, the authors report a nacre-mimetic composite obtained by infiltrating a switchable Diels-Alder network polymer into a scaffold of alumina capable of self-healing internal damage.

## Introduction

Biological structural materials are usually strong, tough, and lightweight owing to their elegant and complex architectures at multiple length scales^1,2^. In particular, the nacreous layer of mollusks, composed of alternating layers of calcium carbonate platelets and biopolymer, exhibits an extraordinary mechanical behavior^3,4^. In the last decade, nacre-mimetic composites (artificial nacres) have been developed using various approaches including magnetic field-assisted additive manufacturing^5^, layer-by-layer assembly^6–8^, spray-casting^9^, ice templating and ceramic sintering^10–12^, matrix-directed mineralization^13^, and evaporation-induced self-assembly^14^. Unfortunately, these artificial nacres are incapable of recovering mechanical damages, a prerequisite for their durability. In addition, while natural nacre exists in intricate forms including spiral and ladle shapes, artificial mimics are limited to simple flat geometries due to the fabrication methods involved^6–14^. Smart nacre with self-healing capability and shape-programmability is highly desirable for practical applications^1–3^, but this demand has not been met.

Self-healing and shape-programmability are commonplace for soft polymers but are rarely realized in rigid structural materials^15–25^. Combining these attributes into a nacreous architecture is challenging: the complex multi-scale structure prohibits the inclusion of encapsulated agents for extrinsic healing^24^; the high loading of rigid fillers restricts the molecular mobility of the soft polymer matrix, a key enabler for intrinsic self-healing and shape adaptability. In principle, the use of dynamic polymer network^25–27^ as the matrix represents a potentially attractive approach to design such a smart nacre. However, harnessing the related properties in a nacreous composite requires reconsideration of molecular mobility from a different perspective due to the physical confinement by the heavily loaded inorganic fillers. We report hereafter our successful attempt in this direction.

Specifically, we are able to incorporate self-healing and shape-programmability into a nacre-mimicking composites by using a dynamic network polymer with high thermal switchability as the polymer matrix. In addition to its capability of healing internal damages, the macroscopic shape of this smart nacre can be programmed in two different ways (permanently via plasticity and temporarily via shape memory effect), in sharp contrast to the intractable planar shape of known artificial nacres. Our study highlights the possibility of harnessing the rich designability of soft polymers in a predominantly inorganic structural material system. The simplicity in the composite fabrication and its scalability imply that various filler systems with functions beyond structural are possible. Therefore, we believe that our approach paves a way for designing durable multifunctional bioinspired structural materials for various practical applications.

## Results

### Fabrication of the smart nacre

**Fig. 1:**
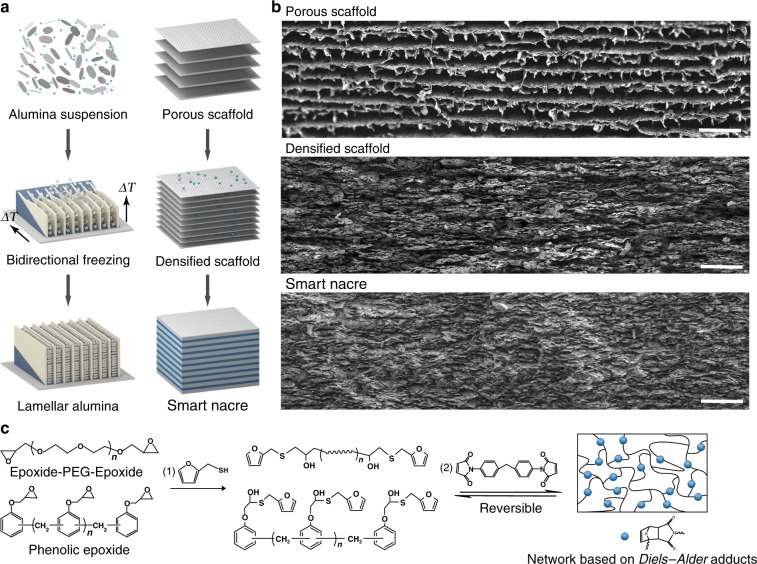
Fabrication of the smart nacre by infiltrating a long-range aligned alumina scaffold with a thermally switchable Diels–Alder network polymer. **a** Fabrication steps for the smart nacre. **b** Scanning electron microscope (SEM) images of the porous alumina scaffold, the densified scaffold, and the smart nacre. **c** Synthetic route and reversible crosslinking for the Diels–Alder network polymer. Scale bar in **b** is 100 μm

Fabrication of the smart nacre. Our smart nacre was fabricated by infiltrating a thermally reversible Diels–Alder network polymer into a long-range-ordered lamellar scaffold of alumina platelets (Fig. 1). Specifically, an aligned porous three-dimensional (3D) scaffold was first obtained by bidirectional freezing^28^ of an alumina slurry (*V*_f_ = 10%) (Fig. 1a). The associated bidirectional temperature gradients in both the horizontal and perpendicular directions were created by placing a polydimethylsiloxane wedge on a cold finger. Upon freezing, alumina platelets were repelled in between the ice crystals. After sublimating the ice, a porous scaffold with a long-range lamellar architecture was obtained. The porous scaffold was pressed and calcined to generate a densified scaffold. The scaffold was then silane-treated to introduce furfuryl surface groups and the dynamic network polymer was infiltrated to yield the smart nacre. The morphologies of the porous scaffold, densified scaffold, and the smart nacre were illustrated in Fig. 1c. More detailed characterizations can be found in Supplementary Figure 1.

The Diels–Alder network polymer was synthesized in two steps (Fig. 1c). First, polyethylene glycol with two epoxide end-groups (XY-225) and phenolic epoxide (DEN 431) were reacted with furfuryl mercaptan. This yielded a furan functionalized oligomer as confirmed by Fourier transform infrared (FTIR, Supplementary Figure 2) and nuclear magnetic resonance (NMR, Supplementary Figure 3) analyses. Next, the oligomer was mixed with a stoichiometric amount of 4,4′-bismaleimidodiphenylethane to produce a liquid precursor. The precursor was thermally cured at 50 °C to form a Diels–Alder network, as verified by a characteristic peak of Diels–Alder adducts at 1775 cm^−1^ in the FTIR spectrum (Supplementary Figure 2). The Diels–Alder network polymer was chosen in order to simultaneously achieve shape memory and healing. While shape memory requires only localized chain mobility, healing requires chain movement at a larger length scale^29–31^. In addition, healing at the filler–polymer interface would also require bond reconnection, which is only possible with dynamic covalent bonds based on such as the Diels–Alder chemistry.

To produce the smart nacre, the Diels–Alder network polymer was infiltrated into the densified alumina scaffold in its liquid precursor form followed by thermal curing. For better comparison, nacre-like composites with less ordered alumina platelets were prepared by the unidirectional freezing method^10,11^ (Supplementary Figure 4). Monolithic samples (45 × 45 × 3 mm^3^) can be obtained (Supplementary Figure 5), reflecting the ready scalability of the process. The energy dispersive spectroscopy (EDS) characterization confirms the successful infiltration of the resin into the scaffold (Supplementary Figure 6). In addition to the ceramic content, the ratio between two epoxide oligomers (XY-225 and DEN 431) has a strong impact on the mechanical properties of Diels–Alder network both at the pure polymer and composite levels (Supplementary Figure 7). The optimum molar ratio of 50/50, corresponding to the best combination of strength and toughness, was used throughout the following study. Owing to the extrinsic toughening at multiple scales resulted from the well-aligned ceramic layers (Supplementary Figure 8), our smart nacre (70 vol% alumina unless otherwise noted) is stiff (bending modulus: 3.6 ± 0.5 GPa), strong (fracture stress: 62.2 ± 5.8 MPa), and tough (toughness: 170.1 ± 10.3 J m^−3^, calculated from the integrated area under stress–strain curve). As unnotched samples were applied in the test, the calculated area represents the upper limit of the work of fracture rather than an accurate estimation^32^. Most strikingly, we illustrate below that the rigid smart nacre with such a high content of inorganic fillers exhibits self-healing capability that is typically reserved for soft polymers.

### Self-healing behavior of the smart nacre

**Fig. 2:**
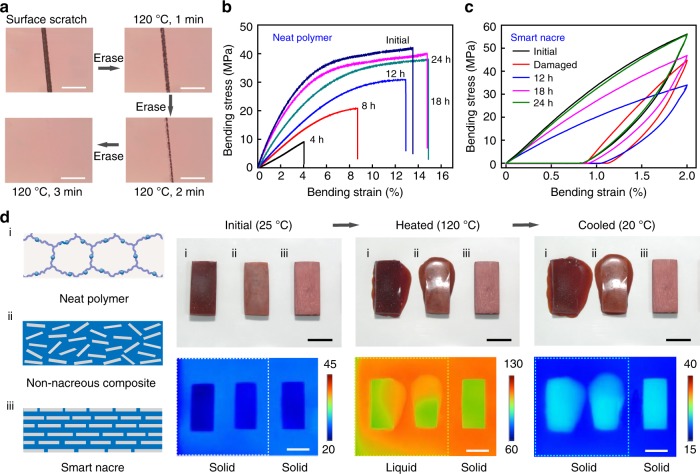
Self-healing behavior of the smart nacre. **a** Sequential optical images recording the erasure of a surface scratch on the neat polymer. Stress–strain curves of the remolded neat polymer (**b**) and self-healed smart nacre (**c**). Note that while the composite was damaged by fatigue test, the healing property of the neat polymer was investigated by cutting a bulk sample into two halves and remolding them via the reversible network formation. **d** Comparisons between the neat Diels–Alder polymer, a non-nacreous composite with random fillers, and the smart nacre with aligned fillers: thermal stability illustrated via optical and infrared images. Scale bar in **a** is 100 μm and in **d** is 1 cm

The healing arises from the reversible nature of the polymer matrix (Supplementary Figure 9)^21,33^. For the neat polymer, disintegrating the Diels–Alder network by heating allows material flow to erase surface scratch (Fig. 2a, Supplementary Movie 1). Similarly, the mechanical performance of a bulk sample cut into two halves can be recovered by remolding via the reversible network formation (nearly full recovery of strength and toughness in 24 h, Fig. 2b). At the composite level, this translates to an intriguing capability to heal internal damages. Specifically, the smart nacre was subjected to a fatigue test (30 cycles of loading/unloading at a bending strain of 2%, which is typical for nacre-mimetic composites^1,3,6–11^ to mimic conditions that a structural composite can experience in real-world engineering applications. The damaged sample showed significantly deteriorated mechanical performance relative to the initial sample (Fig. 2c) despite the absence of any visible flaws. Different from the fracture of the pure polymer, possible damages in the composite are: rupture of polymer network matrix; rupture of the filler/polymer interface; displacement of alumina platelets; all at the molecular scale, regardless of the type of damages (such as cracks, scratches, and breaks). The strain of 1% in the fatigue test is very small for the polymer matrix to rupture. By comparison, rupture of the interfacial filler–polymer bonding is likely due to the drastically different rigidity and weak adhesion at organic and inorganic interface. Within each ceramic layer, the nanoplatelets are surrounded by the polymer. Mechanical loading only changes their relative position without leading to their breakage. We therefore believe that rupture of interfacial bonding and relative platelet displacement are the damages occurred in the tests. With heating (120 °C) alone, the composite gradually regained the mechanical property, reaching complete recovery after 24 h. Similar healing capability was found when tested in the direction parallel to the ceramic layers (Supplementary Figure 10). In consistent with bending tests, the smart nacre is also healable in tensile test (Supplementary Figure 11). Herein, we note that this type of internal damages is commonly expected for structural composites (e.g. carbon fiber composites)^34^. The invisible nature is of particular concern as their accumulation can lead to catastrophic failure without warning. In fact, this represents a major hurdle for wide-spread use of structural polymer composites in situations when safety is most required. Thus, healing invisible internal damages as demonstrated for our smart nacre is of prime importance.

We emphasize that achieving self-healing for the nacreous composite is non-trivial. To probe the mechanism, a neat polymer, a non-nacreous composite with randomly distributed alumina fillers, and a smart nacre are compared (Fig. 2d, Supplementary Movie 2). Upon heating to 120 °C, both the neat polymer and the non-nacreous composite lose their dimension due to the material flow in sharp contrast to the stable dimension for the smart nacre (Fig. 2d). The drastically different dimension stability suggests the important role of the nacreous architecture presumably due to the inherent capillary interaction arisen from the high degree of filler alignment. On the flip side, it implies that the molecular mobility of the polymer matrix is severely reduced due to the physical confinement, unfavorable for healing. We emphasize that both composites in Fig. 2d contains only 10 vol% alumina platelets. The restriction of molecular mobility for the smart nacre in Fig. 2c (with 70 vol% alumina) is expected to be much more severe, making the observed healing behavior particularly intriguing. The reversible nature of the polymer matrix plays a key role. Specifically, it transitions from a solid (Young’s modulus of 0.8 ± 0.1 GPa) to a liquid (viscosity of 50.2 ± 6.5 mPa s, 120 °C) when heated to 120 °C. This low viscosity (high flowability) for the neat polymer is balanced by the nano-confinement (interspacing between the platelet filler is calculated to be around 20 nm) in the smart nacre. The net result is that sufficient molecular mobility at the nanoscopic scale can be achieved while maintaining the macroscopic dimension stability.

### Self-healing mechanism and mechanical recovery behavior

**Fig. 3:**
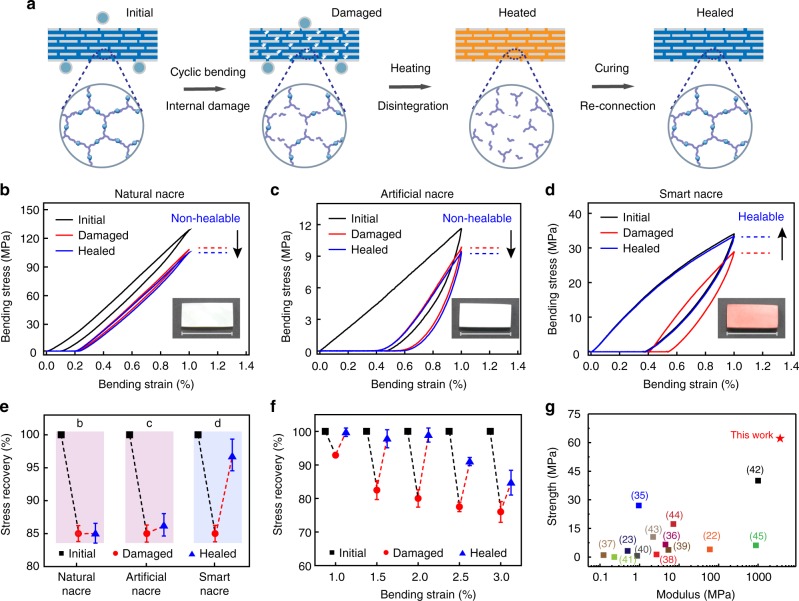
Self-healing mechanism of the smart nacre and its mechanical recovery behavior. **a** Schematic illustration of the damage-healing process of the smart nacre. Stress–strain curves recorded during the damage-healing process for a natural nacre (**b**), an artificial nacre infiltrated with non-healable polymer (**c**), and the smart nacre (**d**). Insets are the optical images for the corresponding samples. **e** Summary of the normalized strength for the initial, damaged, and healed specimens for the three types of samples in **b**–**d**. Summary of the normalized strength for the initial, damaged, and healed smart nacre, when damaged under different bending strain (**f**). **g** Ashby chart summarizing the strength vs. modulus of various self-healable materials. Error bars represent standard deviations calculated from five specimens. Scale bar in **b**–**d** is 2 cm

With sufficient mobility, the reversible Diels–Alder chemistry allows healing internal damages (chain-scissions) in the polymer matrix by reforming the network structure (Fig. 3a). Importantly, this healing mechanism also applies to damages at the polymer–filler interface due to the interfacial Diels–Alder connections. Direct comparison between a natural nacre, an artificial nacre with an irreversible epoxy matrix, and the smart nacre further reveals the essential role of the Diels–Alder network matrix for healing. Damages were first created for all the three samples by cyclic loading at a fixed bending strain of 1% until their strength decreased to 85% of their initial values. They were then heated to 120 °C to promote healing. From their stress–strain curves before and after heating (Fig. 3b–d), it is evident that neither the natural nor the artificial nacre shows any sign for healing in sharp contrast to the near full recovery for the smart nacre. Specifically, for the strength, the smart nacre recovered to 96% of its initial value whereas the other two samples do not show much improvement (Fig. 3e). The healing behavior of the smart nacre is affected by the level of bending strain in the damage experiments. Figure 3f shows that the bending stress can be almost fully recovered for small strains (0–2%). At higher strains, the healing becomes noticeably less efficient but the stress can still recover to more than 80% at 3% strain. Similarly, the healing efficiency is also dependent on the fatigue cycles with longer cycles leading to less stress recovery (Supplementary Figure 12). Specifically, 95% stress recovery is achieved at 30 cycles, which reduces to 80% at 60 cycles.

Indeed, the healing efficiency is strongly related to the size of defect. However, there is no threshold size limit (or turning point) for healing, which is mostly limited by the filler mobility within the polymer matrix. When bending at a strain of 3%, the displacement of the fillers is roughly 15 nm, as estimated by the average in-plane dimension of the fillers (around 500 nm). Such a small defect size makes it nearly impossible to visualize either the damage or repair at the microscopic scale. As the mobility of ceramic fillers is intrinsically low due to the tight packing and relatively large size (comparing to small organic molecules from the Diels–Alder moieties), it is more difficult for the displaced alumina platelets to return back to their original positions. Therefore, the recovery of the overall mechanical property is less efficient when damaged at a larger strain or with more fatigue cycles.

In addition, the strength vs. modulus of various self-healable materials reported in the literature (Supplementary Table 1) are summarized in an Ashby chart in Fig. 3g. With superior strength and modulus than typical soft polymers^22,23,35–45^, our smart nacre opens up new possibilities for designing self-healable rigid materials which are highly demanded for various applications. It should be noted that conventional self-healing composites could be really strong but can be healed only once. This is because their self-healing capability is usually based on an extrinsic mechanism, such as the encapsulation of healing agent within hollow fibers or microcapsules^46,47^. In contrast, our smart nacre is self-healable for multiple times based on an intrinsic mechanism provided by the dynamic covalent polymer network.

### Shape programmability of the smart nacre

**Fig. 4:**
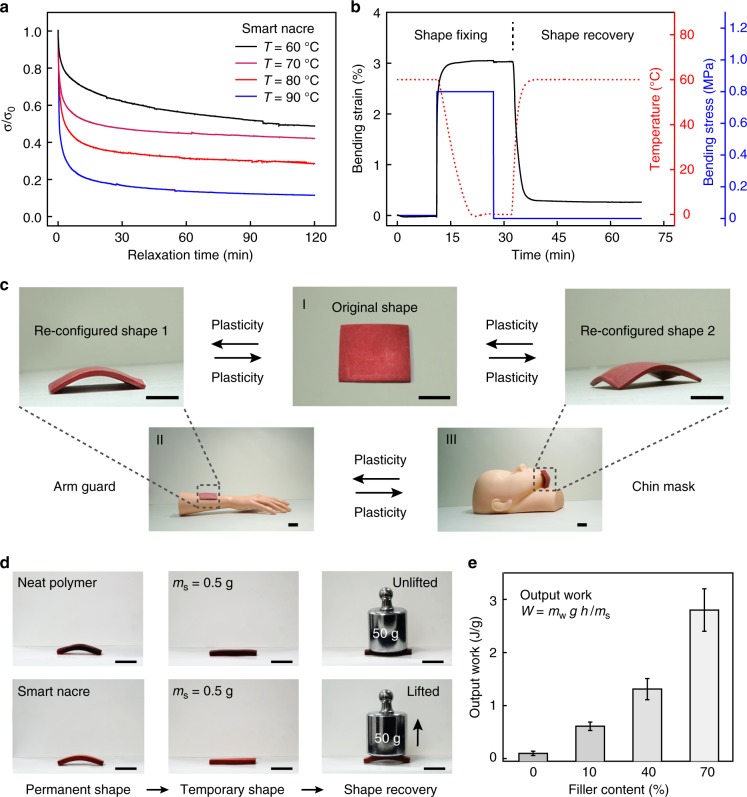
Shape programmability of the smart nacre. **a** Stress relaxation of the smart nacre under iso-strain conditions (3%). **b** Elasticity-based shape memory cycle of the smart nacre. **c** Shape re-configuration via plasticity. **d** Shape recovery under a 50 g load for neat polymer and the smart nacre (*V*_f_ = 70%). **e** Summary of the output work (*W* = *m*_w_ *gh*/*m*_s_) for neat polymer and smart nacres with various filler contents, in which *m*_w_, *g*, *h*, and *m*_s_ represent the load, gravitational acceleration, lifted height, and sample weight, respectively. Error bars represent standard deviations calculated from five specimens. Scale bar in **c** is 2 cm and in **d** is 1 cm

The delicate material design that enables the self-healing can also be extended to surprisingly versatile shape-programmability that is otherwise not known for artificial nacre. Figure 4a illustrates the stress relaxation behaviors of the smart nacre under iso-strain conditions, with faster relaxation kinetics for higher temperature. The behavior arises from the reversible network topological rearrangement and implies the ability to permanently alter the shape by solid-state plasticity^25^. Specifically, a shape retention ratio (*R*_ret_) of 95.2%, which reflects near perfect permanent shape programmability, was obtained after stress relaxation at 90 °C for 120 min (Supplementary Figure 13). The glass transition temperature of the neat polymer is around 35 °C (Supplementary Figure 14). Using this phase transition, an opposite mechanism that allows elasticity-based shape memory behavior is feasible. Accordingly, Fig. 4b shows that the smart nacre can be programmed at 60 °C to fix a temporary shape (strain) upon cooling, with a responding shape fixity (*R*_f_) of 99.0%. Upon reheating under stress-free condition, the original shape (strain) is recovered, with a shape recovery ratio (*R*_r_) of 91.3%. Figure 4c visually shows that an initial planar sample can be permanently programmed via plasticity to yield different functional 3D shapes, indicating potential applications in such as an arm guard and a chin mask. Note that the type of performance requirements can be quite diverse depending on applications. In these two scenarios for potential orthopedic applications, healing capability at 2% strain readily meets the requirements; the fracture strain of commonly used fiber-reinforced plaster composites is typically below 0.5%^48^. Moreover, the healing and programming can be conveniently performed by heating in an oven (120 °C). The shape memory can be conducted at 60 °C with a hair dryer, and brief body exposure to this temperature is not an issue. The temporary shape fixing and recovery performance of the smart nacre is demonstrated in Fig. 4d. Notably, the recovery can be accomplished under a 50 g load. Note that the maximum recoverable strain of the smart nacre is equal to its fracture strain like a typical shape memory material^49^. By comparison, the neat polymer fails to recover under the same load. Figure 4e illustrates that the recovery output work by the smart nacre (2.8 J g^−1^ for filler content of 70%) is significantly enhanced, reaching around 30 times of the neat polymer. A video capturing the recovery under load is provided as Supplementary Movie 3.

## Discussion

In summary, we demonstrate that dynamic covalent polymer network can be utilized as the polymer matrix to enable the fabrication of a smart nacre with intrinsic self-healing and versatile shape-programming capacities. More generally, the study highlights the possibility of harnessing the rich designability of soft polymers in a predominantly inorganic structural material system. Additionally, the simplicity in the composite fabrication and its scalability imply that various filler systems with functions beyond structural are possible. Overall, the study points to a future direction in designing bioinspired structural material systems with multi-functions beyond those typically associated with a single biological material.

## Methods

### Materials

Platelet alumina powder (Rona FlairTM White Sapphire) was purchased from Merck. Sodium dodecyl sulfate (92.5–100.5%) and poly(vinyl alcohol) (*M*_w_ ~ 205,000), 2,4,6-tri(dimethylaminomethyl) (95%), and methylhexahydrophthalic anhydride were supplied by Aladdin. Polydimethylsiloxane (PDMS, Sylgard 184) was bought from Dow Corning. Epoxide oligomers including polyethylene glycol with two epoxide end groups (Trade name: XY-225) and phenolic epoxy (Trade name: DEN 431) were purchased from Anhui Xinyuan Chemical and the Dow Chemical, respectively. Furfuryl mercaptan (98%) was supplied by J & K. 4,4′-Bismaleimidodiphenylethane (>96%) was purchased from Tokyo chemical industry. Trimethoxy[3-(furfurylmethoxy) propyl]-silane was purchased from Shanghai Gileader Advanced Material Technology.

### Preparation of the long-range aligned alumina scaffold

Long-range aligned lamellar alumina scaffolds were fabricated using a bidirectional freezing method. A slurry was first prepared by mixing deionized water with alumina platelet powder (10 vol%), sodium dodecyl sulfate (2.5 wt% to alumina), and poly(vinyl alcohol) (2.5 wt% to alumina) under gentle stirring at 80 °C for 2 h. The slurry was further ball-milled for 24 h followed by sonication for another 2 h. The slurry was degassed in a vacuum desiccator prior to freezing. During the bidirectional freezing, the slurry was poured into a plastic mold with a PDMS wedge at the bottom. The mold was then placed on a cold copper plate connected to a cold source with cryogenic ethanol (−90 °C) for 1 h. Finally, sublimation of the ice at −80 °C (12 Pa for 24 h) resulted in a long-range aligned lamellar scaffold.

### Synthesis of the Diels–Alder polymer network

The two epoxides XY-225 and DEN 431 were mixed at a predetermined ratio. The mixture was then reacted with a stoichiometric amount of furfuryl mercaptan at 60 °C for 3 h under gentle stirring with tris(dimethylaminomethyl) phenol (1 mol% to epoxide) as the catalyst. The resulting liquid mixture were further heated to 120 °C and mixed slowly with a stoichiometric amount of 4,4′-bismaleimidodiphenylethane to generate a liquid precursor. Finally, a Diels–Alder network was obtained after curing at 50 °C for 24 h.

### Preparation of the nacre-mimetic composites

The porous alumina scaffold (*V*_f_ = 10%) with the long-range aligned lamellar structure was vertically pressed (~4 MPa) and calcined at 600 °C for 2 h to remove the emulsifier. The densified alumina scaffold was then modified with furan groups by immersion into a trimethoxy[3-(furfurylmethoxy) propyl]-silane/methanol (3 vol%) solution for 24 h. After the silane treatment, the sample was dried at 60 °C for 2 h. The nacre-mimetic composite was prepared by infiltrating the liquid precursor at 130 °C into the alumina scaffold. The nacre-mimic composite was obtained after thermal curing at 50 °C for 24 h. As a reference, an artificial nacre without the Diels–Alder moieties was made by infiltrating methylhexahydrophthalic anhydride-cured epoxy (XY-225:DEN 431 = 50:50) into the densified scaffold. The inorganic scaffold of the non-nacreous composite with randomly distributed fillers was also modified with furan groups following the same procedure as for the nacre-mimetic composite.

### Characterization

^1^HNMR spectra were recorded using a Bruker 500 MHz spectrometer with CD_2_Cl_2_ as the solvent. Fourier transform infrared spectra were collected using a Nicolet 5700 spectrometer by Thermofisher. Surface healing of the neat polymer was investigated using a laser scanning confocal microscopy (LSM, Zeiss) equipped with a heating stage (MK2000, Instec). SEM images and EDS data were collected on a field-emission SEM instrument with EDS component (SU-3500, Hitachi). Infrared images were taken with an infrared camera (Ti10, Fluke).

Mechanical properties were evaluated by three-point bending and tensile using Instron 5944. Cuboid samples (20 × 2 × 2 mm^3^) were used and the loading/unloading rate was 0.015 mm s^−1^. A minimum five specimens were tested for each sample. Differential scanning calorimetry was conducted using DSC Q200 (TA instruments).

Dynamic mechanical analyses were conducted using DMA Q800 by TA instruments. For measurement of the glass transition temperature, tests were run in “multi-frequency, strain” mode. For evaluation of the plasticity (stress relaxation) and shape memory performance, experiments were carried out in “stress relaxation” and “force control” modes, respectively. The shape retention ratio during plasticity testing was calculated from equation (*R*_ret_ = *ε*_ret_/*ε*_load_), in which *ε*_load_ and *ε*_ret_ represent the strain before and after load removal, respectively. In a shape memory (elasticity) cycle, shape fixity ratio, and shape recovery ratio were calculated as *R*_f_ = *ε*_d_/*ε*_load_ and *R*_r_ = (*ε*_d_ − *ε*_rec_)/*ε*_d_, respectively. *ε*_d_, *ε*_load_, and *ε*_rec_ represent the fixed strain after cooling and load removal, the maximum strain under load, and the recovered strain, respectively.

## Supplementary information

Supplementary Information

Description of Additional Supplementary Files

Supplementary Movie 1

Supplementary Movie 2

Supplementary Movie 3

## Data Availability

The data that support the findings of this study are available from the corresponding authors upon request.
