# Supplementary material for: Nacre-mimetic composite with intrinsic self-healing and shape-programming capability
Source: Nat Commun. 2019 Feb 18;10:800. doi: 10.1038/s41467-019-08643-x (PMC6379389; doi:10.1038/s41467-019-08643-x)
Supplement: Supplementary file 1 — Supplementary Information [file 41467_2019_8643_MOESM1_ESM.pdf]

Supplementary Information

Nacre-mimetic composite with intrinsic self-healing and shape-programming capability

Du et al.

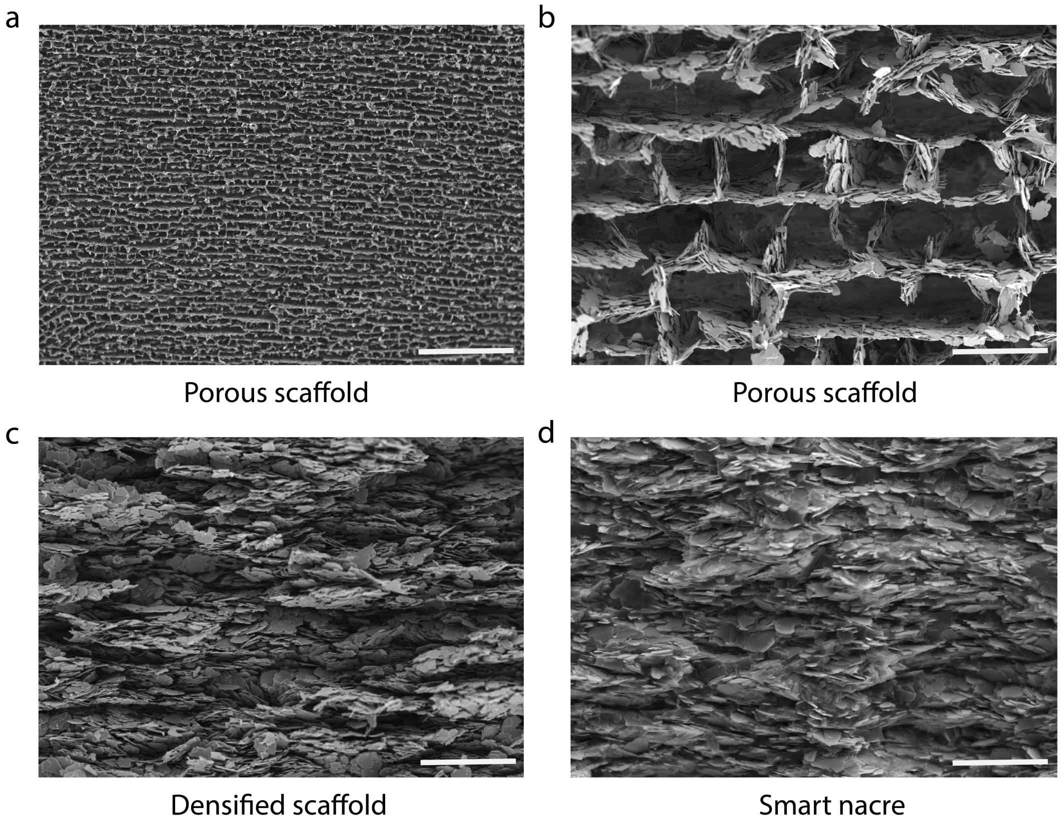

Supplementary Figure 1 | SEM images of the porous (**a**-**b**) and densified scaffold (**c**) of alumina platelets, and smart nacre by infiltrating the densified alumina scaffold with Diels-Alder network polymer (**d**). Scale bars in **a**–**d** are 500, 50, 50, 50 μm, respectively.

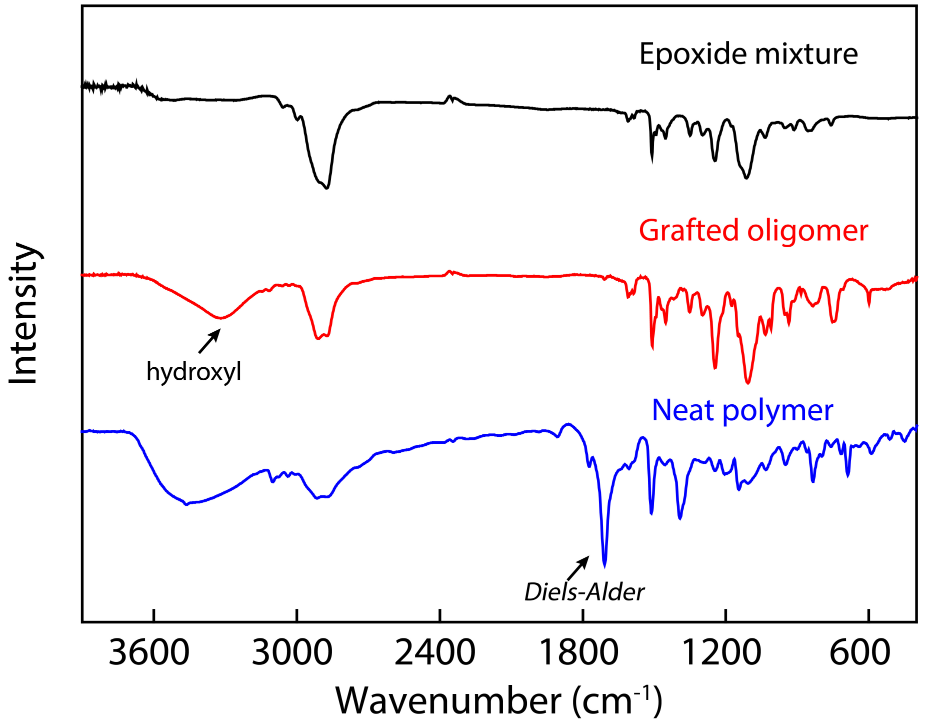

Supplementary Figure 2 | FTIR spectra of the starting epoxide mixture, the furfuryl grafted oligomer (the appearance of hydroxyl groups at 3200 cm^{-1} indicates the occurrence of the grafting reaction), and the Diels-Alder network polymer (the peak of Diels-Alder adducts at 1755 cm^{-1} confirms the crosslinking).

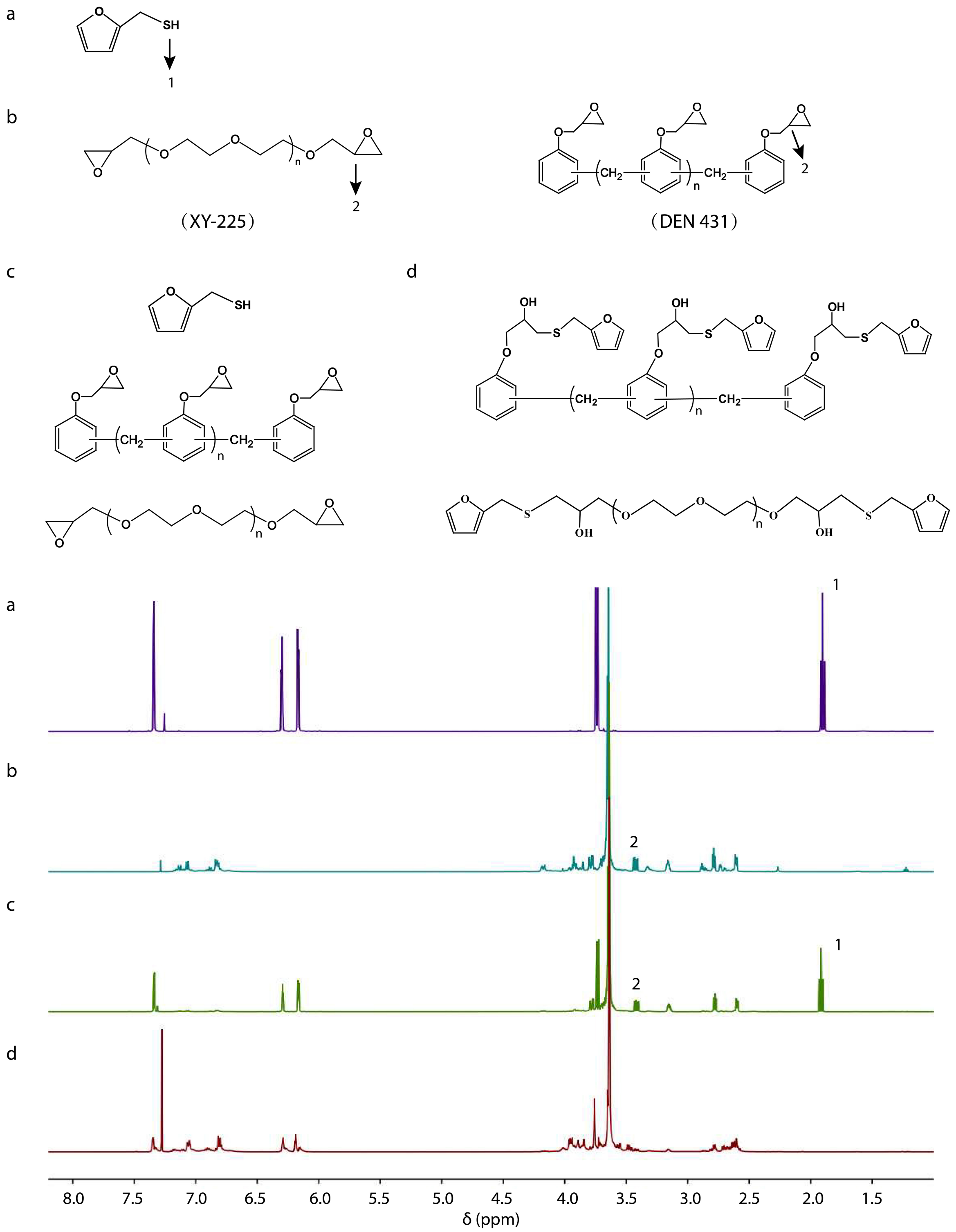

Supplementary Figure 3 | ^{1}HNMR analysis of the precursors for the Diels-Alder polymer. a, Furfuryl mercaptan monomer (peak 1: thiol). **b**, Epoxide mixture (peak 2: epoxy). **c**, Mixture of the furfuryl mercaptan and epoxide (with both specific peak1 and 2 detected). **d**, Reaction product of the furfuryl mercaptan and epoxide (both the peak 1 and peak 2 disappeared, confirming their reaction).

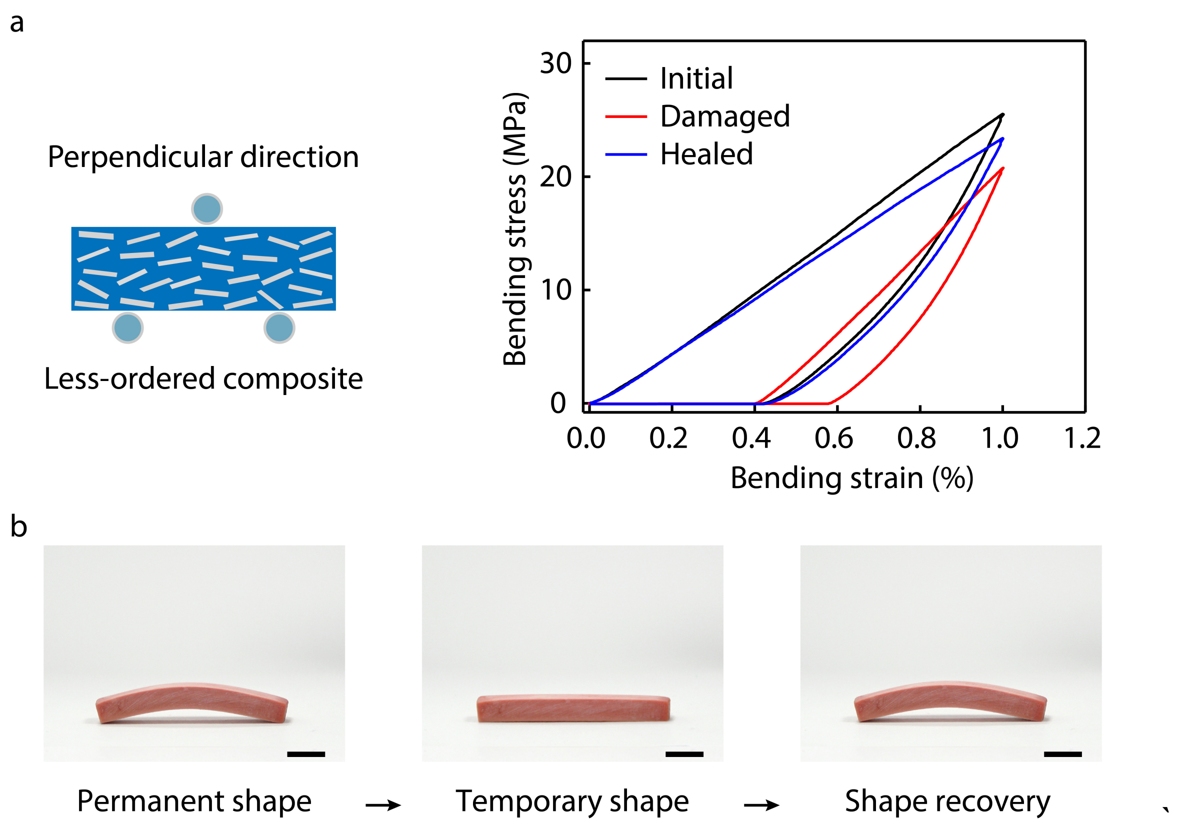

Supplementary Figure 4 | Self-healing and shape recovery capability of the less-ordered nacre-mimetic composite prepared by unidirectional freezing. a, Bending stress-strain curves recorded during the damage-healing process. **b**, Shape recovery process. Scale bar in **b** is 5 mm.

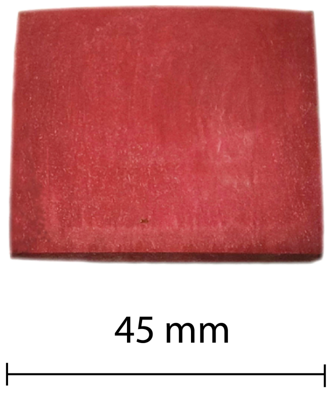

Supplementary Figure 5 | Optical image for a large piece of smart nacre, reflecting the ready scalability of the fabrication procedure.

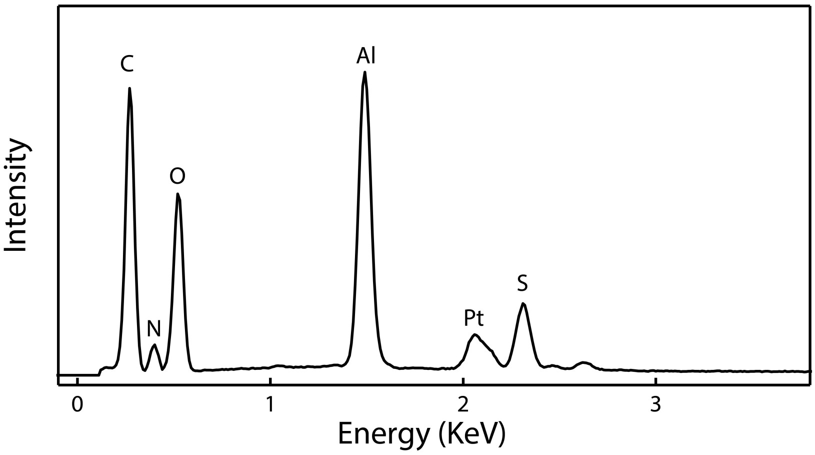

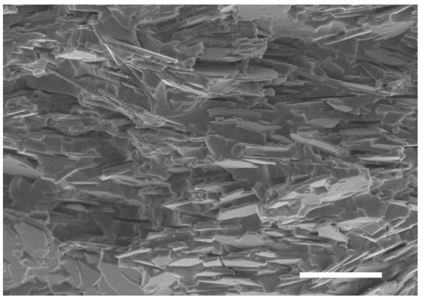

Element	Mass percent (%)	Atomic percent (%)
C	52.9	63.9
N	2.3	2.4
O	27.3	24.7
Al	13.0	7.0
S	4.5	2.1

Supplementary Figure 6 | Energy dispersive spectroscopy (EDS) characterization of the smart nacre. It confirms the successful infiltration of the resin into the scaffold, as indicated by the 'C', 'N' and 'S' elements. Scale bar is 10 μm.

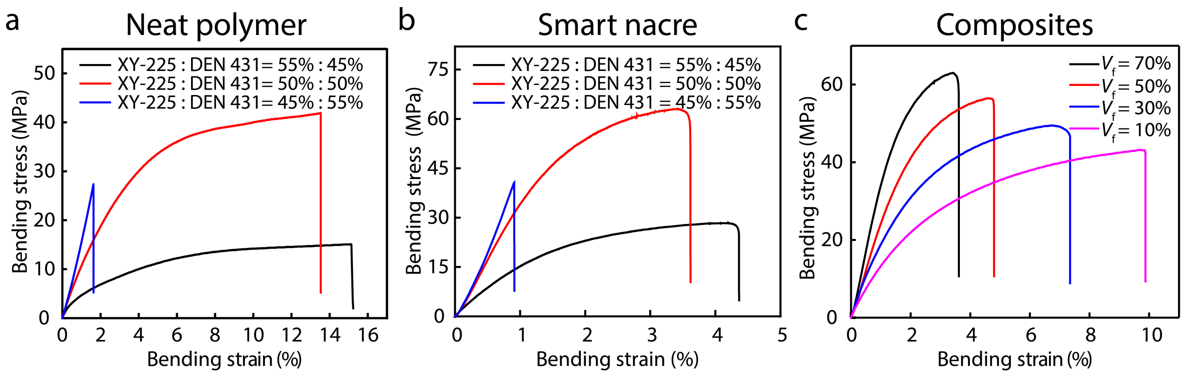

d

Summary of mechanical properties of resins

Polymer composition	Ceramic content (%)	Modulus (GPa)	Strain (%)	Strength (MPa)	Toughness (J/m^3)
45 : 55	0	1.3 ± 0.3	1.5 ± 0.3	24.1 ± 2.5	20.9 ± 1.3
50 : 50	0	0.8 ± 0.1	13.1 ± 1.0	40.2 ± 4.1	435.4 ± 21.9
55 : 45	0	0.5 ± 0.1	15.1 ± 0.6	15.4 ± 1.2	177.7 ± 10.8

e

Summary of mechanical properties of composites

Polymer composition	Ceramic content (%)	Modulus (GPa)	Strain (%)	Strength (MPa)	Toughness (J/m^3)
45 : 55	70	4.7 ± 0.4	1.0 ± 0.07	39.9 ± 2.5	16.6 ± 1.1
50 : 50	70	3.6 ± 0.5	3.4 ± 0.14	62.2 ± 5.8	170.1 ± 10.3
55 : 45	70	1.5 ± 0.3	4.2 ± 0.08	29.1 ± 1.4	90.7 ± 5.4
50 : 50	50	2.4 ± 0.2	4.8 ± 0.21	56.5 ± 3.2	189.1 ± 9.8
50 : 50	30	1.6 ± 0.1	7.4 ± 0.35	49.4 ± 3.5	270.1 ± 12.6
50 : 50	10	1.3 ± 0.1	9.9 ± 0.32	43.1 ± 2.6	309.8 ± 15.1

Supplementary Figure 7 | Mechanical properties of the neat polymer and smart nacre. Three-point bending stress-strain curves for the neat polymer (**a**) and their corresponding smart nacre with different epoxide ratios (XY-225:DEN 431) (**b**) and ceramic content (**c**). The mechanical behavior of the polymer is strongly dependent on the ratio between XY-225 and DEN 431. Their chain rigidity is drastically different with the aliphatic and aromatic chains being soft and rigid, respectively. In addition, each XY-225 bears two reactive epoxy rings, whereas DEN 431 has on average 2.8 epoxy groups. The difference in chain rigidity and number of epoxy functional groups (thus crosslinking density) together contributes to this strong dependence. **d**-**e**, Summary of the mechanical properties depicted in (**a**-**c**).

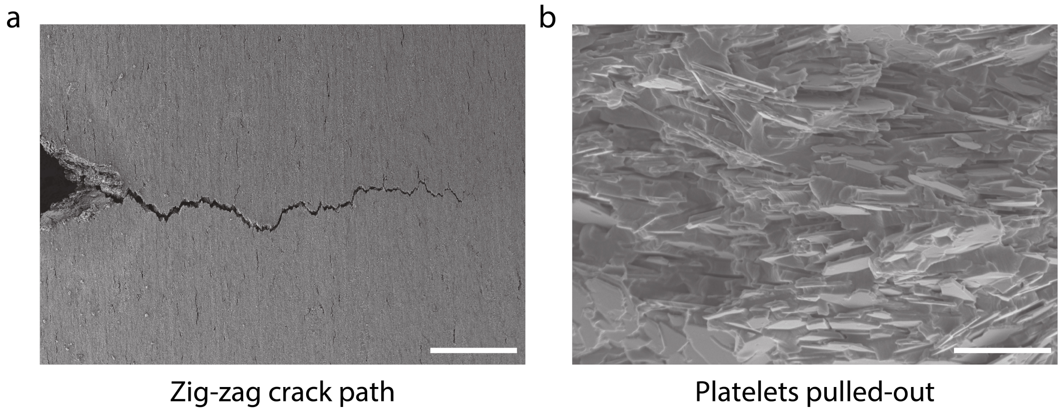

Supplementary Figure 8 | Crack propagation and fracture surface of the smart nacre indicating extrinsic toughening mechanism resulted from the well-aligned ceramic layers. a, The zig-zag crack path for a pre-notched sample under bending test. **b**, Pull-out of platelets at the fracture surface. Scale bars in **a**–**b** are 200, 10 μm, respectively.

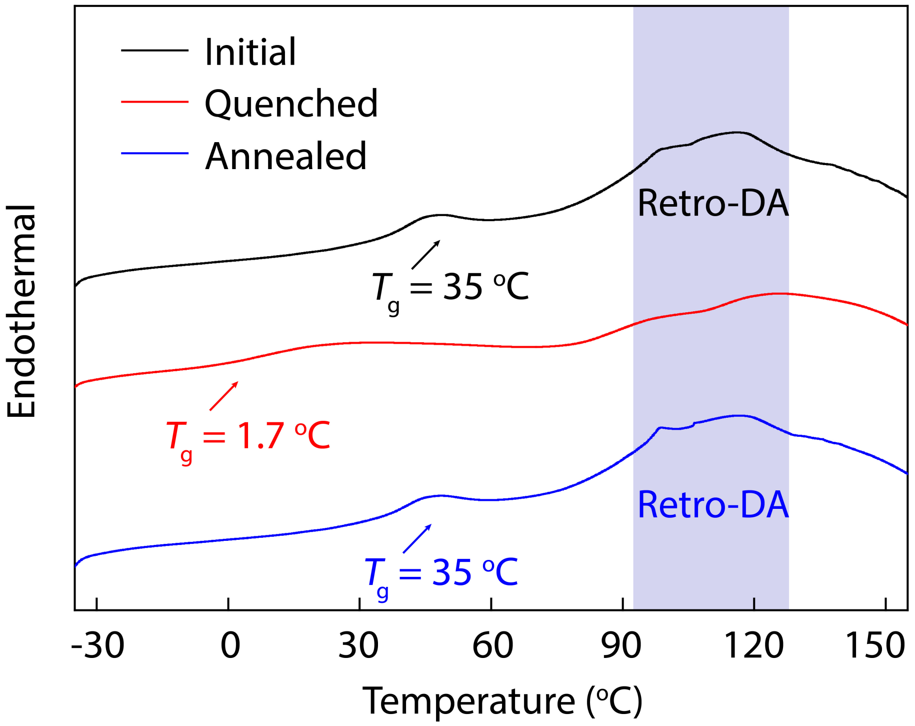

Supplementary Figure 9 | Differential scanning calorimetry (DSC) curves for the Diels-Alder polymer network. The initial sample shows a T_g transition at 35 °C and a peak related to retro-Diels Alder reaction (~100 - 130 °C). Upon heating to disintegrate the network via the retro-Diels Alder reaction followed by quenching, the T_g was reduced to 1.7 °C. Upon further thermal annealing at 50 °C for 24 h, the network sample recovers its T_g and the overall DSC feature is recovered (similar to the initial sample). **Measurement conditions:** Differential scanning calorimetry was conducted using DSC Q200 (TA instruments). The heating rate was 10 °C·min^{-1} and the cooling rate was -30 °C·min^{-1}. The neat polymer sample was first heated from -40 to 160 °C. Then, the heated sample was quenched to -40 °C. Next, the quenched sample was heated to 160 °C and cooled to 50 °C and annealed at this temperature for 24 h before the DSC measurement.

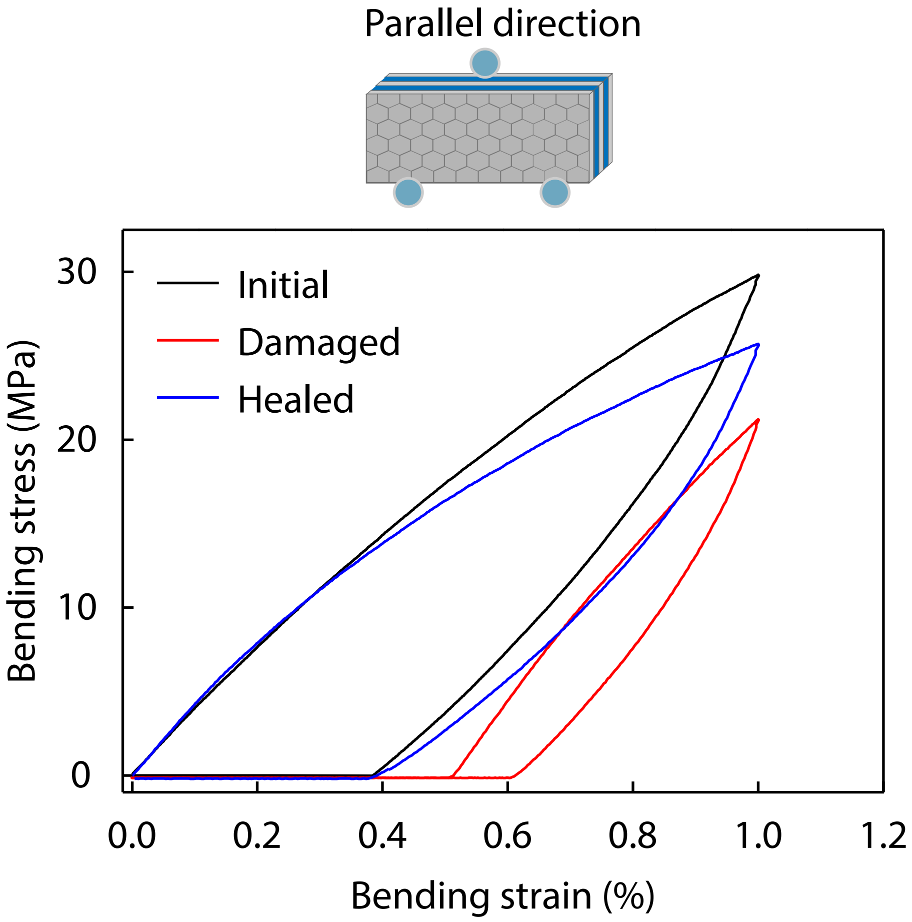

Supplementary Figure 10 | Stress-strain curves recorded during the damage-healing process for a smart nacre tested in the direction parallel to the lamellar layers. The sample was damaged by fatigue test under 1% bending strain for 10 cycles, with its strength decreased to around 71.1% of its initial value. After healing, its bending strength recovered to around 86.2%. Importantly, the modulus and strain are fully recovered, judging from the initial portion of the stress-strain curve. This is in sharp contrast to the behavior prior to the healing.

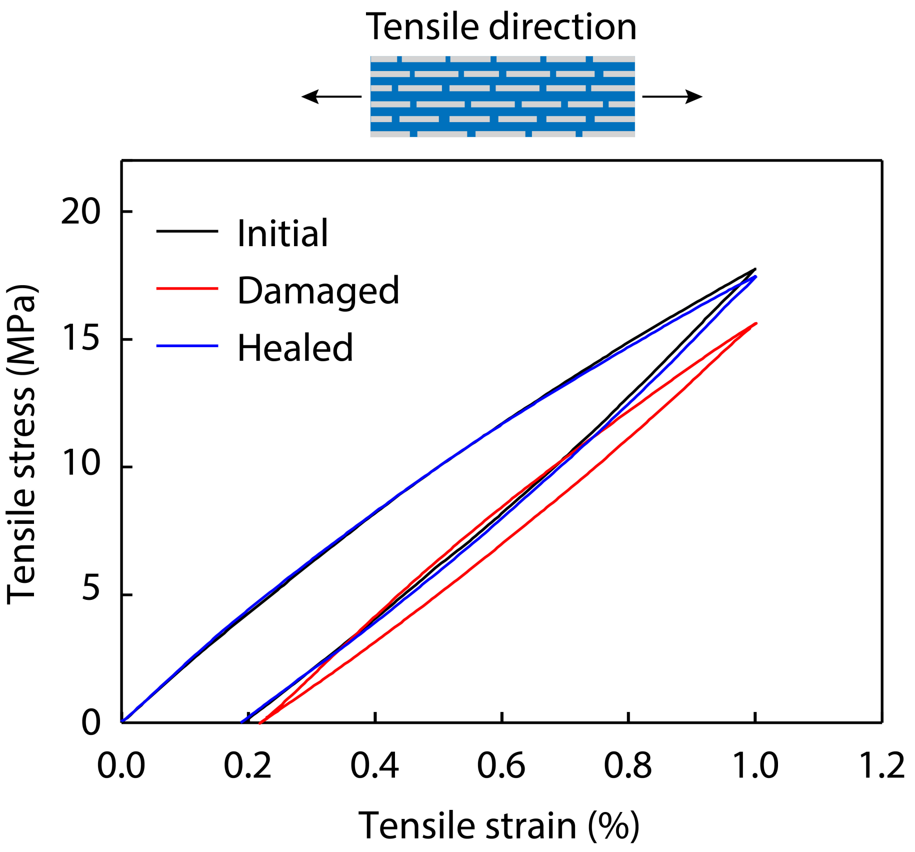

Supplementary Figure 11 | Tensile stress-strain curves recorded during the damage-healing process for a smart nacre. It shows full recovery of the damaged sample, in consistent with the bending tests.

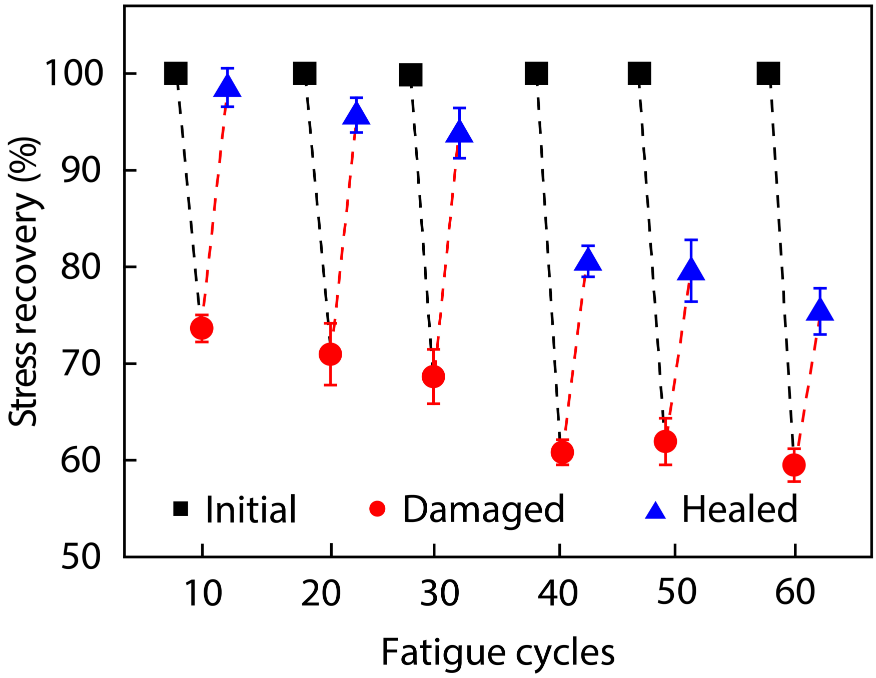

Supplementary Figure 12 | Summary of the normalized strength for the initial, damaged, and healed smart nacre, when damaged under different fatigue cycles by bending. Error bars represent standard deviations calculated from five specimens.

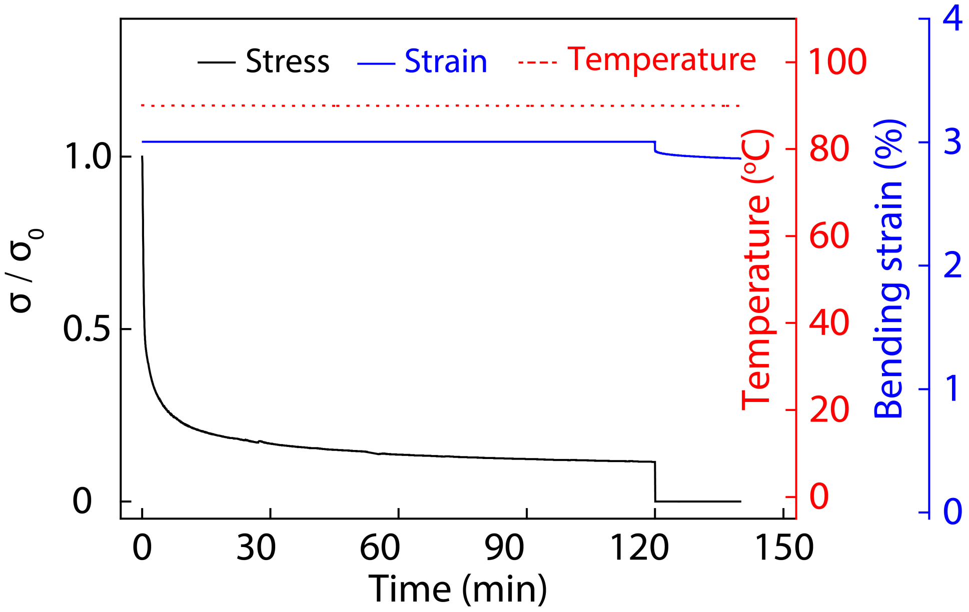

Supplementary Figure 13 | Plasticity based permanent shape reconfiguration behavior of the smart nacre. The smart nacre exhibited significant stress relaxation at 90 °C for 120 min. After the stress relaxation, the stress was completely removed and the corresponding permanent shape retention ratio was calculated as 95%.

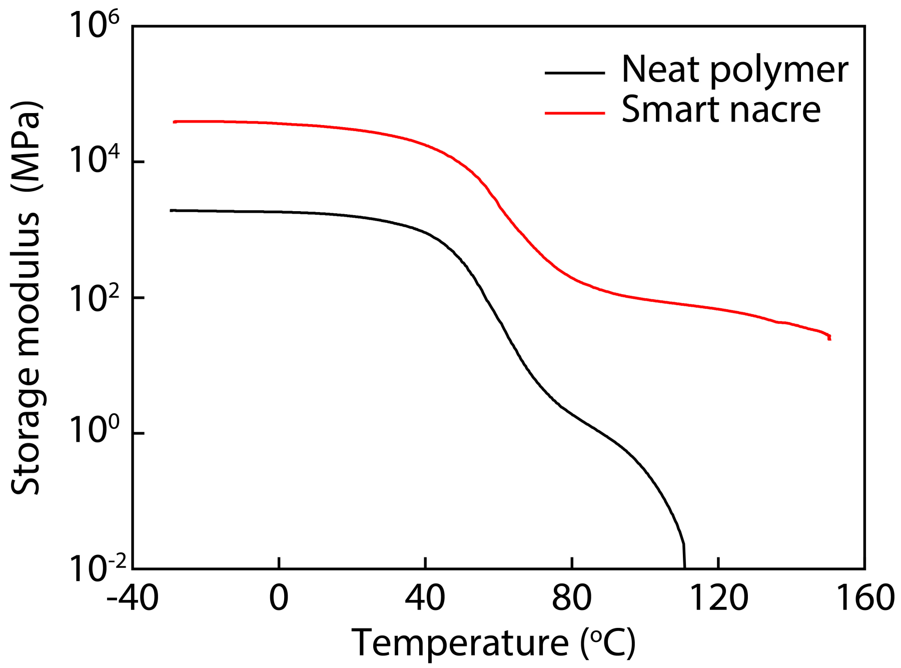

Supplementary Figure 14 | Thermal-mechanical curves of the neat polymer and smart nacre. The glass transition temperatures are similar. However, the neat polymer starts to flow at 110 °C while the smart nacre shows a rubbery plateau due to the nanoconfinement by the aligned fillers.

Supplementary Table 1 Summary of mechanical behavior of various self-healable materials.

	References	Species	Mechanism	Modulus (MPa)	Strength (MPa)	Healing Ratio (%)	Healing Conditions
1	Polymer 52, 6074-6079 (2011)	Resin	*Diels-Alder* reaction	1.0	27	46	100 °C, 5 days
2	Polymer 124, 48-59 (2017)	Resin	*Diels-Alder* reaction	4.5	6.55	71	120 °C, 15 min<br>60 °C, 24 h
3	Adv. Funct. Mater. 25, 1598-1607 (2015)	Hydrogel	Hydrogen bond	0.123	1.0	40	95 °C, 2 h
4	Angew. Chem. 124, 10713–10717 (2012)	Elastomer	Hydrogen bond	2.7	1.35	＞90	45 °C, 18 h
5	Adv. Mater. 27, 2722-2727 (2015)	Hydrogel	Ionic bond	5.4	3.7	66	Contacted together in air for 12 h
6	Nature 451, 977-980 (2008)	Rubber	Supramolecular assembly	0.5	3.2	80	20 °C, 180 min
7	Nature 472, 334–337 (2011)	Rubber	Metal–ligand interactions	60	3-4	100	<195 °C, 60 s
8	Nat. Commun. 5, 3218 (2014)	Resin	Urea bond	0.87	0.62	29	37 °C, 20 h
9	Adv. Mater. 28, 8419–8424 (2016)	Hydrogel	Supramolecular interactions	0.23	0.01	95	37 °C
10	Adv. Mater. 26, 3938–3942 (2014)	Resin	Schiff base	1000	40	100	80 °C, 30 min
11	Adv. Mater. 28, 4884–4890 (2016)	Hydrogel	Ionic bond	2.2	10.5	85	60 °C, 90 min
12	Angew. Chem. 56, 13464 –13469 (2017)	Hydrogel	Hydrogen bond	7.2	17.3	94	65 °C, 30 min
13	Adv. Mater. 29, 1701399 (2017)	Coating	Supramolecular interactions	880	6.1	60	33 °C, 30 min
14	Compos. Sci. Technol. 65, 1791-1799 (2005)	Composite	Epoxy curing (Extrinsic self-healing)	/	733	93	Room temperature, 24 h
15	Smart Mater. Struct. 17, 015019 (2008)	Composite	Epoxy curing (Extrinsic self-healing)	9000	240	34	130 °C, 1 h<br>Room temperature, 24 h
16	This work	Composite	*Diels-Alder* reaction (Intrinsic self-healing)	3600	62.2	>95	120 °C, 10 min<br>50 °C, 24 h
