# Supplementary material for: Nacre-mimetic composite with intrinsic self-healing and shape-programming capability
Source: Nat Commun. 2019 Feb 18;10:800. doi: 10.1038/s41467-019-08643-x (PMC6379389; doi:10.1038/s41467-019-08643-x)
Supplement: Supplementary file 2 — Description of Additional Supplementary Files [file 41467_2019_8643_MOESM2_ESM.pdf]

Description of Additional Supplementary Files

File Name: Supplementary Movie 1
Description: Surface scratch erasing process for the neat polymer.

File Name: Supplementary Movie 2
Description: Confined molecular mobility of polymer within the nacreous architecture.

File Name: Supplementary Movie 3
Description: Shape recovery of neat polymer and smart nacre under a 50 gram load.
